# Designing Gender-Responsive Health Promotion Programs for Men: A Scoping Review

**DOI:** 10.1177/10901981251322391

**Published:** 2025-03-17

**Authors:** Paul Sharp, Caitlin Sankey, John L. Oliffe, Nico Schulenkorf, Cristina M. Caperchione

**Affiliations:** 1 University of New South Wales, Sydney, Australia; 2 University of Technology Sydney, Sydney, Australia; 3 University of British Columbia, Vancouver, Canada; 4 University of Melbourne, Melbourne, Australia; 5 Stellenbosch University, Stellenbosch, South Africa

**Keywords:** men’s health, health promotion, community-based participatory research, gender

## Abstract

Over the past decade, there has been an increased emphasis on tailoring men’s health promotion programs. To optimize outcomes, participatory action research that involves and elicits feedback from end-users has been highlighted as important to creating gender-responsive interventions. In this scoping review, we examine (a) how participatory action research has been used to design health promotion interventions for men and (b) what constitutes a gender-responsive intervention design. Following a comprehensive search, 53 articles were included in the review, reporting on 35 men’s health promotion programs. Our findings suggest that participatory action methods harness varying degrees of end-user involvement, with a large majority limited to post-intervention evaluations rather than co-design and consumer collaboration. In addition, there are inconsistencies for applying gender-responsive approaches within programs, particularly regarding how interventions are targeted, tailored, and promoted to men. We conclude that participatory action research methods translate to varying degrees of gender responsiveness in men’s health promotion programs. That said, involving end-users at various stages of intervention design, implementation, and evaluation may increase the likelihood that programs are more attuned to masculinities and better engage participants in promoting healthy behavior change. Efforts to advance gender-responsive designs can benefit from inductively deriving and incorporating men’s masculine values.

Men have historically been underrepresented in the design and delivery of health promotion interventions across numerous social determinants (e.g., health status, cultural background, ethnicity, age) and health outcomes (Guillermo et al., 2022; Howell et al., 2023; Sharp, Spence, et al., 2020). As a result, the past decade has seen a sharp rise in community-based health interventions targeted at men, with an emphasis on marginalized sub-groups (Bottorff et al., 2015; Gross et al., 2023; S. Robertson & Baker, 2017). Herein, gender-sensitized designs for men have emerged as key to program efficacy (Fleming et al., 2014), with the centerpiece typically being traditional masculine norms (e.g., protector/provider, stoicism, self-sufficiency) that are marketed as pliable drivers for health behavior change (Griffith et al., 2020; Jewkes et al., 2015; Milner et al., 2019; Seidler et al., 2016). While these approaches have garnered some men’s engagement and benefited their health outcomes (Timm et al., 2024), many health promotion programs have been predicated on shifting men’s alignments to traditional masculine norms (Smith & Robertson, 2008). Recent calls have been made for the need to inductively derive potential end-user’s lived experiences and needs to develop more relevant and meaningful gender-responsive offerings (Galdas et al., 2023; Östlin et al., 2007).

Numerous gender-related factors (e.g., perceived societal roles) can impact the uptake of health promotion programs (Bell et al., 2023; L. M. Robertson et al., 2008; S. Robertson & Baker, 2017; Seidler et al., 2016) and there is evidence that interventions tailored to men’s interests increase engagement (Caperchione et al., 2015; Garcia et al., 2022; Seaton et al., 2017; Sharp et al., 2018; Valdez et al., 2021). Gender responsiveness, or the extent to which programs consider gender norms and promote gender equity, is an important consideration when designing and evaluating interventions (Dworkin et al., 2015). To assess this, the World Health Organisation Gender Responsive Assessment Scale (GRAS) was created with the aim of lessening gender-based health inequities (World Health Organisation [WHO], 2011). The scale outlines five categories ranging from gender-unequal to gender-responsive (WHO, 2011). While the GRAS was originally designed to address gender inequity factors in the health outcomes of women and girls (WHO, 2011), the scale has been applied to men’s health research garnering attention to the role of masculinities and the need for programs to address unproductive masculine norms (Dworkin et al., 2015; Flood, 2019; S. Robertson et al., 2013; WHO, 2011).

In shifting away from gender-mainstreaming approaches which fail to address contextual diversities between genders, gender-sensitive approaches initially emerged as good practice (Fleming et al., 2014). Several authors have developed design tools (Struik et al., 2019), program principles (Oliffe et al., 2020), and process development frameworks (Galdas et al., 2023) to guide and advance gender-sensitized men’s health promotion programming. Struik et al. (2019) evaluated the Check-Mate tool, which considered five key approaches for planning, implementing, and evaluating men’s health promotion programs. Factors included creating man-friendly spaces, incorporating appealing activities for men, using masculine ideals to increase the wellbeing of men and their families, considering aspects of men’s identities other than gender, and encouraging independence and participation (Struik et al., 2019). The authors cautioned against reinforcing traditional masculine norms, and highlighted the importance of promoting programs as user-led and owned with men as exert co-creators (Struik et al., 2019). Similarly, Oliffe et al. (2020) explored the complexities of men’s health promotion highlighting gendered caveats and contexts. Herein, men’s buy-in was aided by facilitating them to establish their own group-based masculine norms that legitimized collective self-health work (Oliffe et al., 2020). However, there has been growing evidence for the use of gender-transformative approaches that not only acknowledge but actively seek to change gender norms, roles, and relations (Galdas et al., 2023). For example, the 5C framework has been suggested as a way to help standardize the methods by which gender-transformative approaches and masculinities are incorporated and applied across a broad range of settings (Galdas et al., 2023). The framework is comprised of five main development phases (i.e., co-production with stakeholders, program cost, context, content, and communication with participants), informed by gender-transformative approaches (Galdas et al., 2023). Despite the availability of gender-responsive guidelines, the extent to which these practices and principles have been adopted within men’s health promotion programming remains unclear.

Key to gender-transformative approaches is the use of participatory action methods that involve men and relevant stakeholders to guide intervention development and design (Galdas et al., 2023; Vargas et al., 2022). These methods seek to foster collaboration, generate content, and deliver meaningful outcomes by responding to men’s health needs and experiences (Constantin et al., 2022). Moreover, participatory action methods may also be important to understanding and addressing broader social determinants (e.g., SES) that contribute to men’s health inequities (Griffith, 2024; Hankivsky & Hunting, 2022). The use of participatory action methods has been associated with increased health literacy as well as strengthening the overall success of health promotion initiatives (Freire et al., 2022; Greenhalgh et al., 2016; Vargas et al., 2022). While some variability exists in its operational definition, participatory action approaches are user-centered and aim to engage specific groups (e.g., young men) to assist in the development, implementation, evaluation, and dissemination of interventions (Constantin et al., 2022; Freire et al., 2022). Recently, co-design approaches have emerged as core participatory methods, varying in participant involvement from basic consultation, active collaboration, to near-total consumer control (Constantin et al., 2022). Vargas et al. (2022) further differentiates the co-design approach as an active and equal partnership between stakeholders to create solutions to issues previously identified in the intervention planning. Within this context, numerous methods for gathering feedback from participants may be used (e.g., focus groups, interviews, workshops) at various stages in the intervention process (i.e., ideating, creating, refining, implementing, evaluating, sharing; Vargas et al., 2022).

Despite recent guides and principles for designing men’s health promotion programs (e.g., Galdas et al., 2023; Struik et al., 2019), there is little synthesis of programs to distill what constitutes gender-responsive programs, and the role of participatory action methods in tailoring those interventions to men. Accordingly, we conducted a scoping review to examine (a) how participatory action methods are used to design men’s health promotion interventions and (b) what constitutes gender-responsive health promotion programs for men.

## Methods

This review utilizes the Preferred Reporting Items for Systematic Reviews and Meta-Analyses (PRISMA) guidelines and has been prospectively registered in the Open Science Framework registry (OSF registration osf.io/7p8x9).

### Eligibility Criteria

Health promotion programs broadly included interventions that actively engaged participants in health-related behavior change (e.g., physical activity, healthy eating, social connection) to improve one or more health outcomes (e.g., weight loss, mental wellbeing). Interventions may be delivered in a range of contexts and settings and that targeted sexual and/or oral health were not within the scope of this review. There were no restrictions on study design (e.g., randomized controlled trial [RCT], pre-post, interviews, mixed-method design, focus groups, etc.) provided they met the following criteria: (a) the intervention or program focused on men’s health promotion, (b) the research was conducted to identify a need and/or inform intervention development for men, (c) intervention participants were adult (18+ years) men. Mixed-gender studies were excluded as gender-blind interventions have long been the norm which so often failed to engage men (Oliffe et al., 2020; Sloan et al., 2010). In addition, it is uncommon that mixed-gender studies include and/or describe gender-tailored design elements (Sharp et al., 2022). Articles published in English from 2013 to 2023 were considered to reduce the number of eligible studies and reflect current trends and directions in the literature. No gray literature or reviews were included.

### Search Strategy

Relevant articles were identified through six electronic databases including Medline, EMBASE, CINAHL, SportDiscus, PsychINFO, and Web of Science. The search strategy (Appendix) was developed using key terms from previously identified literature and Medical Subject Headings (MeSH) terms related to: *men, health promotion, men’s health, lifestyle intervention, wellbeing promotion, community-based participatory research, co-design, action research*, and *gender-responsive design.* The strategy was developed for Medline, and the syntax was adapted across databases. References of included articles were also searched to ensure relevant articles were not missed and to reduce reporting bias. The search was conducted in April 2023 and restricted to studies published within the last 10 years. Covidence was utilized for duplicate removal, screening, and data extraction. Screening was conducted independently by two authors (CMC, CS) and any discrepancies or conflicts were resolved by a third author (PS).

### Data Extraction and Synthesis

Two authors (PS, CS) independently extracted relevant data from the 53 articles, including publication details, participant information, intervention characteristics and development, level of involvement of end-users, and gender-responsive design. Where there were multiple papers for the same program, the information from each paper was collated. Intervention design and delivery features related to the co-design, recruitment, context, content and delivery, and communication style were extracted from available sources (i.e., publications, supplementary materials, program websites) and used to determine gender responsiveness, adapted from the 5C Framework (Galdas et al., 2023). Gender responsiveness was classified using the WHO Gender Assessment Scale as gender-discriminatory (upholds gender inequality), gender-blind (ignores gender expectations), gender-sensitive (considers gender expectations), gender-responsive (considers gender expectations and addresses specific needs), or gender-transformative (includes strategies to promote gender equality and amend harmful expectations) programs (Department of Gender, Women and Health, 2011). Disagreements were reviewed by a third author (CMC) and discussed during bi-weekly team meetings.

A narrative approach was used to synthesize data from the included articles regarding program characteristics, methodological quality, and WHO Gender Assessment Scale classification. The information was thematically analyzed by two authors (PS, CS), and generated keywords from which emerging codes and overlying themes were identified.

## Results

### Study Selection

**Figure 1.**
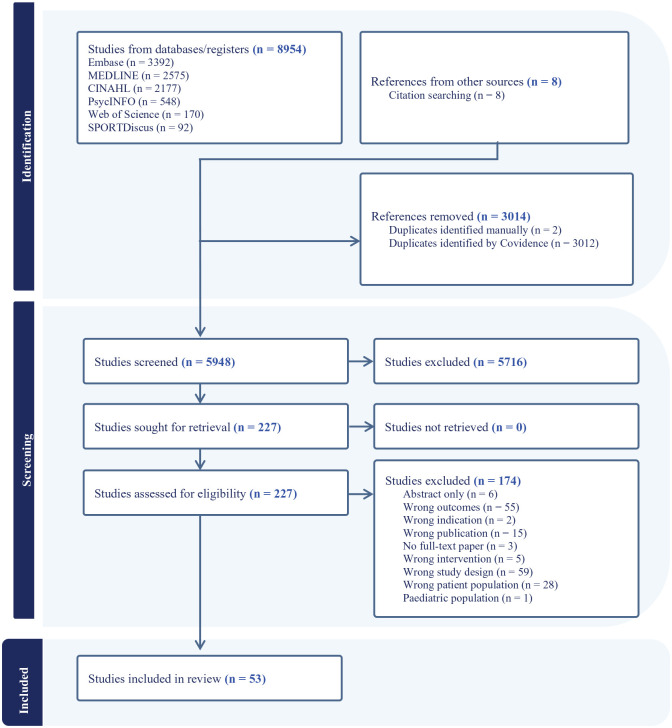
Flow Diagram of Included Articles.

A total of 8,954 articles were retrieved during the initial search. Title and abstract screening was conducted to exclude articles that did not meet the eligibility criteria and remove duplicates. A total of 227 articles were identified for full-text review wherein 174 articles were excluded (e.g., interventions that included female participants). Fifty-three articles were identified for inclusion in the final review to make up a total of 35 programs (see Figure 1).

### Study Characteristics

All included studies were published from January 2013 to April 2023, and the programs were representative of numerous countries, including the United States (*n* = 8; 23%), United Kingdom (*n* = 7; 20%), Australia (*n* = 6; 17%), Canada (*n* = 4; 11%), New Zealand (*n* = 3; 9%), Ireland (*n* = 3; 9%), Denmark (*n* = 1; 3%), India (*n* = 1; 3%), Scotland (*n* = 1; 3%), and Sweden (*n* = 1; 3%). Study designs encompassed pilot studies (*n* = 13; 25%), protocols (*n* = 9; 17%), qualitative designs (*n* = 7; 13%), RCTs (*n* = 6; 11%), quasi-experimental designs (*n* = 6; 11%), mixed methods (*n* = 5; 9%), descriptive designs (*n* = 4; 8%), process evaluations (*n* = 2; 4%), and secondary analyses (*n* = 1; 2%).

### Intervention and Co-Design Methods

The level of end-user involvement varied with the majority of studies involving men in a consultation (*n* = 16; 46%) or collaboration (*n* = 13; 37%) capacity. Only 3 programs (9%) were identified as being consumer led. Within this, the most common stage of development to engage participants was in evaluating (*n* = 28; 80%) the intervention or resource (e.g., through surveys, interviews). Forty-nine percent (*n* = 17) involved participants before intervention implementation (i.e., ideating, creating, refining) and only 9% (*n* = 3) of programs included participants in all five stages of development (i.e., ideating, creating, refining, implementing, evaluating, sharing). Many programs (*n* = 13; 37%) involved participants at only one stage of development. Programs which were collaborative and consumer-controlled were often iterative in their design, flexibly delivered, and provided bespoke offerings tailored to the unique interest and preferences of participants. Notably, all studies were supported, in-part, by limited-term grant funding provided by government, university, and/or non-profit funding that likely limited the longevity and sustainability of many programs. Herein, some authors discussed the challenges of securing ongoing funding and reliance on volunteers (e.g., Kelly et al., 2019). Football Fans in Training (FFIT) provided one model which has been replicated internationally, whereby partnership with and delivery through professional sports organizations has allowed for sustainable delivery. Study and co-design characteristics are further detailed in Table 1.

**Table 1.** Intervention and Development.

Intervention	Country	Study design	Target Pop.	Intervention description	Stage of development	Engagement activities/measurement	Level of involvement of end-users	Funding source
1. Aussie-FIT	Australia	[1] Protocol (Quested et al., 2018) [2] Pilot (Kwasnicka et al., 2020)	Middle-aged men (35–63 years), overweight/physically inactive (BMI ≥28 kg/m^2^), from AFL fan bases (Dockers, Eagles).	The program consisted of 12 weekly 90-minute face-to-face sessions that included physical activity, nutrition, behavior change information, and practical activities.	Creating Refining Evaluating	Survey, interviews	Consultation	Western Australian Health Promotion Foundation (Healthway). Development and Evaluation funded by the Medical Research Council (MRC).
2. Becoming active	New Zealand	Qualitative (Warbrick et al., 2020)	Māori men (28–50 years), sedentary/overweight (BMI ≥ 25 kg/m^2^; exercise <30 minutes twice p/wk), completed the previous intervention with at least 75% attendance.	The program featured a 12-week “culturally enhanced” exercise intervention, consisting of three 30- to 35-minute sessions per week.	Ideating Evaluating Sharing	Focus groups	Consultation	Health Research Council of New Zealand
3. Black Impact	USA	Pilot (Joseph et al., 2023)	Black American men, poor or average cardiovascular health (<4 Life’s Simple-7 metrics in the ideal range).	Due to the suspension of in-person research in March 2020, a 9-week virtual community engagement (VCE) phase was introduced to keep men involved in the Black Impact study. This virtual option was suggested by participants as a way to remain engaged while awaiting the resumption of the in-person intervention, as described in Nolan et al., 2023.	Ideating Creating Refining Implementing Evaluating	Meetings	Consumer control	Ohio State University’s Connect and Collaborate grant; the National Center for Advancing Translational Sciences
4. Black Impact	USA	Descriptive (Nolan et al., 2023)	Black American men, poor or average cardiovascular health (<4 Life’s Simple-7 metrics in the ideal range).	The program was a 24-week community-based lifestyle intervention focusing on physical activity, health education, and addressing social needs. Its components included: –A 24-week intervention covering physical activity, nutrition, and education. –Assistance in navigating participants without a primary caregiver to establish care with a provider. Efforts to address social needs that create barriers to wellness.	Ideating Creating	Community consultation, design workshop	Collaboration	Ohio State University’s Connect and Collaborate grant; the National Center for Advancing Translational Sciences
5. EuroFIT	UK	[1] RCT protocol (van Nassau et al., 2016) [2] Mixed methods protocol (van de Glind et al., 2017)	Overweight men (30–65 years, BMI ≥27 kg/m^2^).	A lifestyle intervention targeting physical activity, sedentary time, and dietary behaviors. 12 weekly, 90-minute sessions combining classroom discussions and graded physical activity in a football club setting. One follow-up meeting 6–9 months after the end of the program.	Evaluating	Focus groups, interviews, questionnaires	Consultation	European Union’s Seventh Framework Program
6. Farmers Have Hearts	Ireland	Protocol (van Doorn et al., 2022)	Irish males (≥18 years), livestock farmers.	The program included two health checks, conducted at baseline and Week 52, and offered a health behavior change intervention with three delivery methods. Participants could choose health coaching by phone, which involved six sessions over 9 months, or mobile health via text messaging, which consisted of 3–4 messages per week for 4 months. They also had the option to combine health coaching and mobile health, with both methods running concurrently. Alternatively, participants could opt for the “usual care” group, which included only the health checks and data collection.	Evaluating	Questionnaires	Consultation	Teagasc, South East Technological University Ireland, Irish Heart Foundation, Health Service Executive and Glanbia Ireland.
7. FC Prostate Community Trial	Denmark	[1] Pilot (Bruun et al., 2014) [2] RCT (Bjerre et al., 2019)	Male prostate cancer patients.	Football (intervention) group: 6 months of recreational football, 1-hour twice a week. Usual care (control) group: 15- to 30-minute telephone session on physical activity options and free-of-charge rehabilitation delivered by the municipalities (this is standard practice in Denmark).	Ideating Creating Refining Evaluating	Interviews, focus groups	Collaboration	TrygFonden
8. FIT4YAMs	Australia	Qualitative (Bailey et al., 2018)	Young adult males (18–25 years), overweight or obese (BMI ≥ 25 kg/m^2^, waist circumference >94 cm), living in a rural center (Bathurst).	2x 60-minute, semi-structured focus groups conducted with six participants. They explored participant preferences for the structure and delivery of a text message-based weight loss intervention.	Ideating Creating	Focus groups	Collaboration	Society for Adolescent Health and Medicine through an unrestricted educational grant from Merck
9. FITShop	USA	Qualitative (Hood et al., 2015)	African-American men, barbershop customers.	FITShop (Fitness in the Shop). A two-phase intervention study to develop (Phase I) and test (Phase II) the effects of a physical activity intervention. Participants were provided with: 1. A pedometer to self-monitor their exercise, physical activity recommendations, personal feedback; 2. Barber workshops and promotion of discussion around exercise between customers and barbers; 3. Barbershop physical activity contests, including culturally targeted educational materials; 4. Referrals to local free or low-cost resources for health and exercise.	Ideating Refining Evaluating	Interviews (owners, barbers, customers), focus groups (customers), Barbershop Advisory Board meetings (did not describe)	Collaboration	National Cancer Institute
10. Football Fans in Training (FFIT)	Scotland	[1] Mixed methods (Gray, Hunt, Mutrie, Anderson, Leishman, et al., 2013) [2] Pilot (Gray, Hunt, Mutrie, Anderson, Treweek, & Wyke, 2013) [3] Process evaluation (Hunt et al., 2020)	Men (35–65 years), overweight or obese (BMI ≥ 27 kg/m^2^).	Comprised of weekly 90-minute sessions over 12 weeks. Each session included: (a) classroom-based education (e.g., healthy eating, reducing alcohol intake, increasing daily exercise), and (b) coach-led physical activity (e.g., aerobic, strength, flexibility) tailored to individual fitness levels, abilities, and pre-existing health conditions. Sessions were complemented by an incremental, pedometer-based walking program.	Refining Evaluating	Focus groups, surveys (participants and coaches), interviews, feed back and training workshop (coaches)	Consultation	Chief Scientist Office and SPL Trust (Study 1, 2). UK National Institute for Health Research Public Health Research (NIHR PHR) Program, UK Medical Research Council.
11. Football in the Community (FitC)	UK	Qualitative (Curran et al., 2016)	Men (18–45 years), living in homeless shelters and/or recovering from substance misuse.	12-week physical activity intervention using football to target men in hard-to-reach populations. The intervention involved a weekly 2-hour physical activity (i.e., football) session, delivered alongside healthy lifestyle information.	Evaluating	Ethnographic methods (e.g., informal conversations)	Consultation	National Cancer Institute
12. HAT TRICK	Canada	[1] Protocol (Caperchione et al., 2017) [2] Mixed methods (Sharp, Bottorff, et al., 2020) [3] Quasi-experimental (Caperchione et al., 2021)	Men (≥35 years), overweight or obese (BMI > 25 kg/m^2^, pant waist size >38”), engage in <150 minutes of physical activity per week.	12-week intervention to enhance physical activity, healthy eating, and social connectedness in men. Comprised of weekly 90-minute, in-person group sessions.	Refining Evaluating	Questionnaire, interviews, facilitator debriefing sessions	Consultation	Canadian Cancer Society
13. He Pikinga Waiora	New Zealand	Quasi-experimental (Oetzel et al., 2020)	Māori men (BMI ≥ 25 kg/m^2^), at risk for Type II Diabetes and related conditions (e.g., cardiovascular disease, obesity).	The intervention was a 12-week lifestyle program using a single-group pre-intervention/post-intervention design and was implemented in two sequential cohorts. The first cohort, which had limited participants, was redesigned into the second cohort. Cohort 1 included physical activity through four self-selected activity groups, nutrition with weekly 1-hour didactic sessions, and met three times per week for 1-hour sessions. It featured monthly prizes, a Facebook group, and information booklets. Cohort 2 offered individually tailored consultations for physical activity and weekly 30-minute nutrition education. Participants’ meeting frequency was determined by their own preferences. This cohort also included a health screen with a nurse and a GP referral if necessary.	Ideating Creating Refining Implementing (in Cohort 1) Evaluating	Surveys, interviews, meetings/community consultations	Collaboration	Ministry of Business, Innovation and Employment (NZ) Healthier Lives National Science Challenge
14. Heart of Hypertension	USA	Mixed methods (Savoca et al., 2013)	African-American men (18–22 years).	This 6-week intervention involved weekly sessions on exercise, healthy lifestyle education, and meal planning. An individual coaching session for dietary change was also proposed. This preliminary plan was presented to participants and experts/outside advisors (two focus groups), then revised based on their feedback (e.g., obtaining input from participants on the selection of exercise activities). This revised intervention was then tested with a focus group.	Ideating Creating Refining	Focus groups, questionnaires	Collaboration	University of North Carolina Greensboro, School of Human Environmental Sciences
15. HEYMAN	Australia	Pilot (Ashton et al., 2017)	Young adult men (18–25 years), do not meet the national fruit and vegetable intake or physical activity recommendations.	HEYMAN (Harnessing E-health to enhance Young men’s Mental Health, Activity, and Nutrition). Was a 3-month healthy lifestyle program to improve eating habits, activity levels, and overall wellbeing. Weekly 1-hour face-to-face sessions (11x group based, 1x individual).	Ideating Creating Evaluating	Surveys, focus groups	Collaboration	Hunter Medical Research Institute (HMRI)
16. Hockey FIT	Canada	[1] Protocol (Gill et al., 2016) [2] Pilot (Petrella et al., 2017)	Male hockey fans (35–65 years), overweight or obese (BMI ≥ 28 kg/m^2^)	Hockey FIT is an adaptation of Football Fans in Training (FFIT) tailored to a Canadian context. The program consists of 12 weekly 90-minute sessions, which include classroom-based teaching on behavior-change techniques and information on physical activity and healthy eating, as well as exercise sessions featuring aerobic, strength, and flexibility exercises that align with participants’ passion for hockey. This is followed by a 40-week maintenance phase and a 6-month reunion/booster session. Additional support tools include the HealtheSteps app for tracking and sustaining exercise, and the Hockey FIT social network, an online platform customized for each site to connect group members and coaches.	Evaluating	Surveys, interviews (coaches and participants), focus groups, questionnaires	Consultation	Movember Foundation (Canadian Men’s Health & Wellbeing Innovation Challenge)
17. Kerala Smoking Cessation	India	[1][2] RCT (Jayakrishnan, Mathew, et al., 2013, Jayakrishnan, Uutela, et al., 2013)	Men (18–60 years), current daily smokers.	The program included individual counseling sessions over a 6-month period, with sessions scheduled at 2–4 weeks, 4–6 weeks, 3 months, and 6 months after the baseline survey. These sessions focused on developing coping skills, reduction strategies, and stress reduction methods for quitting tobacco, each lasting 15 minutes. In addition, group counseling and medical sessions featured a 20-minute documentary on tobacco and cancer, messages from a local Malayalam actor, and literature on substance abuse and its societal implications. Group interventions emphasized the benefits of quitting tobacco, common withdrawal symptoms, measures to overcome them, and coping and relapse prevention strategies. A general medical camp was also included.	Sharing	Not reported	Not reported	Not reported
18. MAN vs FAT Football (MVFF) Program	UK	Pilot (Budden et al., 2020)	Overweight men (BMI ≥27.5 kg/m^2^).	14-week, weekly weight loss program centered on involvement in a small-sided football league. Before each match, participants were weighed-in and rewarded with a weight loss “goal” if they lost weight in the preceding week. Further off-field goals for reaching various other weight loss milestones were included. Participants also received weekly brief consultations with a league weight loss coach, the use of player handbooks, and communication forums.	Evaluating	Interviews	Consultation	Sport England Lottery and UK Active Awards
19. Meaning-Centered Men’s Groups	USA	Pilot (Heisel et al., 2020)	Men (≥55 years), plan to retire within the next 2 years, within the process of retiring, or retired in the last 5 years, able to acknowledge feelings around their retirement transition.	A 12-session psychosocial group intervention designed to enhance mental wellbeing and prevent or reduce suicide ideation among men struggling with, or concerned about, their transition to retirement. Comprised of weekly 90- to 120-minute community-based group sessions.	Evaluating	Evaluation forms, interviews	Consultation	Movember Foundation (Men’s Mental Health grant)
20. Men on the Move (Ireland)	Ireland	Quasi-experimental (Kelly et al., 2019)	Sedentary men (≥18 years), do not meet national physical activity guidelines, likely to be at risk of cardiovascular disease.	Free 12-week program composed of twice-weekly 1-hour structured group exercise (cardiovascular fitness, strength, and conditioning) along with health-related workshops (mental wellbeing and diet).	Not reported	Not reported	Not reported	Health Services Executive, the Local Sports Partnerships, the Irish Heart Foundation and the Men’s Development Network. Partners with National Partnership Network and local community organizations.
21. Men on the Move (USA)	USA	Pilot (Griffith et al., 2014)	Men who self-identified as African American or Black (≥35 years).	Men were assigned to a small group based on times they identified they could meet. Total of six workout groups, each involved a 1.5-hour physical activity workout, once a week for 10 weeks.	Evaluating	Questionnaires, open-ended feedback (written or verbal)	Consultation	American Cancer Society, the Cancer Research Fund of the University of Michigan Comprehensive Cancer Center, the Cancer Epidemiology Education in Special Populations, and the University of Michigan Center on Men’s Health Disparities
22. Mind Resilience Program	UK	Pilot (Robinson et al., 2015)	Unemployed men (45–60 years).	The Mind Resilience Program included practical activities (e.g., gardening, drumming) in a structured, group setting aimed at increasing men’s sense of wellbeing and social connections while reducing social isolation. Participants received a core resilience coping strategies course which was flexibly delivered and tailored to individual and group needs.	Refining Evaluating	Surveys, interviews	Consultation	Not reported
23. MOCHA Moving Forward	USA	Protocol (Valdez et al., 2021)	Low-income adult black men.	The MOCHA intervention offers two versions: the original model and a narratively enhanced version called MOCHA+ Stories Matter. The standard MOCHA program spans 10 weeks, involving small group discussions on issues pertinent to men of color, health classes focusing on chronic disease control, and twice-weekly moderately intensive aerobic exercise sessions. Both versions emphasize increasing physical activity, with participants encouraged to engage in 60 minutes of moderately vigorous physical activity twice weekly, utilizing local YMCA facilities and guided classes led by trained mentors. MOCHA+ Stories Matter employs interactive techniques like digital stories and narratives to facilitate engagement, social interaction, and personalisation of stress management and chronic disease prevention strategies within the curriculum.	Ideating Creating Refining Implementing	Interviews, workshops.	Consumer control	Not reported. Part of Building Resilient Communities funded by mind.org.uk and the Mental Health Foundation
24. Offload Rugby League Program	UK	Qualitative (Wilcock et al., 2021)	Men who experienced mental illness, and who were at risk of suicide.	The program included 10 weekly sessions which comprised two 40-minute halves that replicated the length of a professional rugby league game. The “first half” included a mental fitness session, and the “second half” involved a range of interactive activities.	Creating	Interviews	Collaboration	Commissioned by Rugby League Cares with funding from the Big Lottery
25. Power Up for Health	USA	Pilot (Gary-Webb et al., 2018)	Men in disadvantaged neighborhoods.	The Power Up for Health program was adapted from the National Diabetes Prevention Program (NDPP) 2012 curriculum to include a year-long program, with 16 weekly sessions, followed by monthly maintenance sessions.	Ideating Refining Implementing Evaluating	Focus groups, interviews	Collaboration	New York State Health Foundation and the National Institutes of Health.
26. POWERPLAY	Canada	[1] Protocol (Caperchione et al., 2015) [2] Pilot (Johnson et al., 2016) [3] Quasi-experimental (Caperchione et al., 2016)	Men in male-dominated workplaces.	POWERPLAY was a workplace intervention that included two 6-week challenges targeted at physical activity and healthy eating.	Implementing Evaluating	Interviews	Collaboration	Canadian Cancer Society Research Institute
27. Premier League Health (PLH)	UK	[1] Quasi-experimental (Pringle et al., 2013b) [2] Qualitative (Pringle et al., 2013a)	Male supporters with heightened health risks, and hard-to-engage men not using primary care and health information services	Premier League Health (PLH) was a nationally-based healthy lifestyle program delivered through 16 English Premier League football clubs. Interventions were flexibly delivered to meet the specific needs of potential recruits and led by Health Trainers, allied health professionals, and staff employed by the clubs.	Implementing Evaluating	Interviews	Collaboration	Football Pools
28. QuitNow Men	Canada	Quasi-experimental (Bottorff et al., 2016)	Adult men interested in quitting smoking.	QuitNow Men is a website with interactive resources and information designed to help men reduce and quit smoking.	Ideating Creating Refining Evaluating	Focus groups, surveys, interviews	Consultation	Research funded by Canadian Cancer Society QuitNow is a province-wide Lung Association quit support resource, funded by the BC Ministry of Health
29. Rugby Fans in Training (RUFIT NZ)	New Zealand	[1] Protocol (Maddison et al., 2020) [2] RCT (Maddison et al., 2023)	Overweight men.	RUFIT-NZ was a 12-week healthy lifestyle program, consisting of 12, 2-hour sessions. Each intervention session included a 1-hour workshop-based education component and 1-hour group-based, but individually tailored, exercise training session.	Evaluating	Interviews	Consultation	Health Research Council Project Grant
30. SHED-IT	Australia	[1] RCT (Morgan et al., 2013) [2] Process evaluation (Morgan et al., 2014) [3] Descriptive (Blomfield et al., 2014) [4] Secondary analysis RCT (Young et al., 2015)	Men (18–65 years), overweight or obese (BMI 25–40 kg/m^2^).	SHED-IT (Self-Help, Exercise and Diet) is a self-administered weight loss intervention for men which included a resource package, consisting of (a) the 25-minute SHED-IT Weight Loss DVD for Blokes; (b) the Weight Loss Handbook for Blokes and the Weight Loss Support Book for Blokes; and (c) a pedometer, tape measure for waist circumference, and a kilojoule counter book. The online group received a website user guide and feedback emails about their food and exercise diaries. The Weight Loss Maintenance Program was complemented the original Weight Loss Program by providing men with additional knowledge, skills, and support in the 6 months immediately post-weight loss.	Evaluating	Surveys	Consultation	(1,2,3) National Heart Foundation Grant-in-Aid. (4) Hunter Medical Research Institute’s Healthy Lifestyles Grant
31. Sheds for Life	Ireland	[1] Qualitative (Bergin & Richardson, 2021)[2] RCT (McGrath et al., 2022)	Adult men (typically older adults).	Sheds for Life was delivered over 10 weeks in a Men’s Shed and included a health check, weekly physical activity sessions (walking or chair-based strength and mobility exercises), weekly healthy eating sessions for 6 weeks and a mental health workshop.	Ideating Creating Implementing Evaluating	Focus groups, interviews	Consumer control	Health Service Executive Ireland and by the Irish Men’s Sheds Association
32. SHIFT	UK	[1] RCT (Clemes et al., 2022) [2] Mixed methods (Guest et al., 2022)	UK HGV truck drivers.	The intervention included an interactive, group-based 6-hour education session delivered by two trained facilitators at the drivers’ workplace. Participants were given a Fitbit Charge 2 and individually tailored “step count challenges” throughout the 6-month intervention. They also received equipment for a “cab workout” in their trucks. In addition, participants were supported by health coaches from the research team via text messages and by trained worksite champions.	Evaluating	Focus groups, interviews	Collaboration	National Institute for Health Research (NIHR) Public Health Research program. The NIHR Leicester Biomedical Research Center. Funding for intervention costs (e.g., Fitbits, cab workout equipment) was provided by the Higher Education Innovation Fund, via the Loughborough University Enterprise Projects Group
33. Sons of the West	Australia	[1] Descriptive (Deans, 2021; Vassallo et al., 2021)	Adult men.	Sons of the West included 10-weekly, 2-hour sessions consisting of an educational presentation and physical activity. The physical activity component offers exercise groups of varying intensity, structured to cater for differing fitness levels and physical abilities.	NR	NR	NR	Victorian Health Promotion Foundation (VicHealth), the Western Bulldogs Community Foundation, and Victoria University
34. Truckin’ Healthy	Australia	Mixed methods (Sendall et al., 2016)	Male truckers (16–69 years).	Workplaces implemented 3–4 interventions over a 6-month period, with the specific choices determined by the workplace manager based on factors such as capacity, time, cost, availability of drivers, and drivers’ preferences. The project team collaborated closely with workplace managers to negotiate and plan the implementation of these interventions. To support this process, the project team provided resources, such as prepared posters, PowerPoint slides, and health messages, along with practical guidance to ensure the successful execution of the chosen interventions.	Ideating Creating Refining Implementing Evaluating	Interviews, surveys, focus groups	Collaboration	Queensland Government under the Healthier. Happier. Workplaces Initiative.
35. ViSiT	Sweden	Pilot (Skagerström et al., 2021)	Men (35–65 years), BMI > 28 kg/m^2^.	ViSiT included 12-weekly group sessions, each consisting of a 1-hour theoretical lecture followed by 30 minutes of physical activity. Activities featured both cardio and resistance training, such as brisk walks and workouts in the stadia fitness center or gym.	Evaluating	Focus groups	Consultation	Not reported

### Gender Responsiveness

Using GRAS definitions (WHO, 2011), 34% (*n* = 12) of interventions were determined to be gendered-transformative, 37% (*n* = 13) gender-responsive, 23% (*n* = 8) gender-sensitive, and 6% (*n* = 2) gender-blind. However, there was significant heterogeneity in the use of gender-responsive terminology, as well as the application of gendered elements within and across studies related to intervention recruitment, context, content and delivery, and communication. Among gender-transformative programs, differences arose in how interventions sought to promote gender equity and address unproductive gender norms. Two-thirds of the interventions used sport and/or physical activity as the backdrop to engage men (*n* = 23; 66%), with 12-week group-based interventions being the most common model for men’s health promotion programs (*n* = 13; 37%). Gender-transformative approaches predominantly utilized group discussion to unpack socio-cultural influences. Herein, the combination of lifestyle and social change helped to facilitate conversations about gender through the use of metaphors as well as a currency for trading social capital (e.g., FITShop, HAT TRICK, Offload Rugby League Program). While some interventions leveraged men’s interest in these activities (e.g., sport fans) for recruitment and engagement, others used this as a platform for promoting social change. That said, few programs directly targeted health-related gender norms, instead focusing more broadly on gender socialization (e.g., masculine identities, peer relationships). Surprisingly, two interventions were deemed to be gender-blind as gender did not feature as an intervention consideration (despite including men-only samples). Table 2 provides an overview of key design features used to determine gender responsiveness.

**Table 2.** Key Design Features Used to Determine Gender Responsiveness.

Intervention	Recruitment	Context	Content and delivery	Communication	Gender responsiveness
1. Aussie-FIT	Men were recruited via weekly fan emails from participating clubs, word of mouth, the Aussie-FIT website, and social media posts from both clubs and Aussie-FIT pages.	Delivered in 2 AFL club facilities.	The program focused on dietary and physical activity changes aligned with masculinized behaviors, such as pairing salad with steak, engaging in football with family, addressing alcohol consumption, weight loss, portion control, and physical activity, using physical items like sandbags and dumbbells to reinforce weight loss. Group support was also encouraged. Coaches shared behind-the-scenes stories and insider knowledge about the club to enhance program delivery.	Leveraged the appeal of AFL to engage overweight and obese Australian men. Unlike previous weight loss programs, this design was tailored to the Australian context and culture, promoting self-regulated behavior control and focusing more on habit formation. The delivery style was informal, fostering positive social interaction, humor, and “friendly banter” to ease discussions on sensitive topics. Reminder text messages were crafted to support men’s autonomy, competence, and connection, encouraging program attendance.	Gender-responsive
2. Becoming Active	Recruited from the previous intervention. Not otherwise described.	Delivered in either a conference room at Massey University in Palmerston North or at a community center in Hamilton, New Zealand.	All exercise sessions were facilitated by Māori men. The intervention utilized common exercise approaches and settings, while incorporating Māori values, principles, and processes.	The research team constantly emphasized that being Māori was a strength and advantage, rather than cause of illness or weakness. Guiding kaupapa principles underpinned all interactions between participants, researchers, and trainers (e.g., emphasis for trainers and researchers to apply manaakitanga (principle related to reciprocation and acknowledging skills/abilities of participants)). This ensured prior knowledge that participants already had of their own health/capabilities was valued.	Gender-responsive
3. Black Impact	Participants from the Black Impact study in [2].	Delivered online through virtual video-conferencing meetings.	Consisted of weekly 1-hour virtual video conference sessions, with the option to increase to 2 hours after Week 5 (i.e., 1 hour of large group sessions, 1 hour of smaller/team-based sessions). In Week 5 (at the request of participants), participants could co-facilitate weekly 1-hour small group calls. Participants were also invited (in 3 of the 9 large group sessions) to facilitate a portion of the session.	The research team utilized culturally tailored messaging to address mistrust of healthcare institutions among Black men. Small groups were created to foster a safe space for discussing mental health, building trust, leveraging spirituality, and forming a sense of brotherhood. Participants encouraged each other by sharing support for weekly session attendance, offering health and exercise tips, and navigating the challenges of being Black men facing systemic racism.	Gender-transformative
4. Black Impact	Participants were recruited from the annual National African American Male Wellness Agency (AAMWA) walk and health fair.	Participants were assigned to teams based on their proximity to a central meeting location (e.g., recreation center).	The intervention was adapted from the Diabetes Prevention Program in addition to the AHA Check, Change, Control Program. A health coach (i.e., physicians, nurses) was assigned to each team and was responsible for implementing the Black Impact curriculum. Key responsibilities included delivering education and establishing and monitoring goal progress. Participants received a 1-year gym membership to a local park and recreation center upon program completion.	NR	Gender-transformative
5. EuroFIT	Recruited from 15 top professional football clubs across the Netherlands, Norway, Portugal, and the UK through online promotions (club/fan websites, social media), emails, newsletters, posters/flyers, match-day advertising, face-to-face recruitment at home games (e.g., handing out leaflets, collecting contact details), local and national media coverage, word of mouth, and the active participation of local supporters’ organizations.	Delivered at club stadiums and/or training facilities to provide an “insider” perspective, enhancing symbolic and physical proximity to the club and fostering a stronger sense of connection and belonging.	The program was delivered by community coaches from professional football clubs, trained to create a positive social environment that supported men in making changes aligned with their preferences and routines. By leveraging male fans’ loyalty to their club, the program attracted participants and offered an opportunity that felt non-threatening to male identities. It encouraged behavior change in ways that challenged but did not conflict with traditional masculine roles, such as learning new skills to take control of their lifestyle. The program was informed by sociological theory, exploring how masculinity and gendered identities intersect with health behaviors in various contexts.	Positive banter was encouraged to foster a supportive team environment, maximizing participant interaction to promote relatedness and sustainable behavior changes around masculine identities and their expression. Participants were encouraged to identify with “men like them” based on similarities in body shape, fitness, and shared interests. The program also promoted the formation of new identities, such as becoming an “active person,” linking masculinities with healthier behaviors and more positive associations.	Gender-transformative
6. Farmers Have Hearts	Posters, engagement efforts by staff of the NGO partner (i.e., invited/encouraged farmers to participate). Recruitment for the baseline health checks was coordinated by the agri-food business (e.g., interactive online portal, text messaging, information leaflets).	Health checks were conducted local workplace venues for farmers (e.g., agri-branch, livestock mart) by nurses working for the NGO. All nurses and health coaches were trained in gender-sensitive approaches to engage farmers. Health coaches also had previous health promotion experience and completed the Engage National Men’s Health Training.	Group 1 (health coaching) was conducted by health coaches. Health coaches also conducted the introductory call for group 3 (combination) and took note of preferred topics for text messages. Gender-specific strategies included: meeting farmers in a safe space, identifying clear health needs, working with rural masculinities, providing factual information, offering choice, and adopting a personal approach. This was combined with strengths-based strategies (i.e., tailored program, non-judgemental, open to all farmers, focused on achievable changes, participant-centered approach) and farmer-centered strategies (i.e., meeting farmers on “their turf,” flexible program elements, farm as health promotion setting, local settings and staff, accounting for farming seasons, outside “office” hours).	All program elements were “non-judgemental” (i.e., accounted for individual health behavior choices thereby respecting participants’ autonomy over their health). The delivery methods were also flexible, practical, and time convenient to allow for the unpredictability of farming. Participants could self-select on their intervention type (i.e., health coaching, mobile, combination) and domain of change (i.e., diet, physical activity, stress management, alcohol consumption). The health coaches were not privy to participant health check results so could not direct participant choice of domain. The mobile health intervention used “everyday” words, active verbs, and short sentences. Each message included the option to opt-out.	Gender-responsive
7. FC Prostate Community Trial	The national prostate cancer organization provided the trial information to its members and provided details about participation through newsletters. Participants were recruited from the Copenhagen Prostate Cancer Center and Department of Oncology at Copenhagen University Hospital.	The intervention was delivered at a local football club.	The intervention aimed to align with traditional masculine ideals as prostate cancer patients can be at risk of loss of masculinity. Delivery was facilitated by coaches from local football clubs who had undergone a 12-hour training seminar on prostate cancer and the intervention manual.	UG participants received an email at the conclusion of their phone session containing the same information. Both participants and coaches had access to an online portal that constituted a 2-way intervention and enabled personalized feedback to participants and continuous evaluation of the intervention progression to coaches. At the end of the 6-month period, FG participants were able to continue the intervention by joining the football club on the local club’s terms.	Gender-sensitive
8. FIT4YAMs	Participants were recruited using traditional print media (e.g., flyers, advertisements) and face-to-face approaches.	Focus groups were facilitated by an interviewer, not otherwise described.	Discussion centered around a highly personalized intervention program (including personalized goal setting, motivation, text message content, and engagement strategies), minimal direct contact with the intervention team beyond establishing brief initial contact (but with clear guidelines and reminders of key contacts should they need help). In addition, a comprehensive goal setting and personalisation session prior to the intervention was discussed as beneficial.	The interviewer provided periodic verbal summaries of the participants’ responses for confirmation purposes and to ensure any dissenting viewpoints were heard. The interactive group setting allowed participants to react and build on others’ responses.	Gender-sensitive
9. FITShop	Recruited from four local barbershops. Implied that this was done through the print materials and mirror cling messages given to barbershops, but unclear.	A barbershop setting was used to deliver the program, traditionally visited by men.	The intervention, delivered by the research team with support from male shop owners and barbers, followed a social ecological approach across intrapersonal, interpersonal, organizational, and community levels. Key elements included: (a) personalized physical activity feedback and pedometers for self-monitoring (intrapersonal); (b) barber workshops to encourage physical activity discussions between barbers and customers (interpersonal); (c) physical activity contests with culturally tailored educational materials (organizational); and (d) referrals to free or low-cost local health resources (community). Participants aimed for 10,000 steps per day using pedometers.	Print materials were distributed to participating barbershops with educational messages on topics such as “get moving” (exercise initiation tips), “holiday moving” (exercising during holidays), “New Year moving” (adapting to new habits), “strong moving” (strength training and safe exercising), and “keep moving” (sustaining exercise). Barbershops also received monthly mirror clings with key messages to prompt conversations between barbers and customers. Participants received tailored feedback reports comparing their current exercise levels to recommended guidelines, along with personalized tips to boost self-efficacy, motivation, and reduce barriers to physical activity.	Gender-transformative
10. Football Fans in Training (FFIT)	Advertised the program on club websites, loudspeaker announcements at home matches, other online advertising, leaflets to season ticketholders. The program was also picked up by local and national media, newspapers, and other third parties.	Delivered by SPL club community coaches (mostly male sessional or fulltime coaches). A female coach supported her male colleague in one club, and a few engaged external male health trainers to support their staff in delivery.	The program was delivered at the club home stadia by 11 of Scotland’s top professional SPL football clubs. Included components designed to appeal to male football supporters, including: club-based incentives (e.g., t-shirts in club colors), competition elements (e.g., quizzes), and a session focusing on alcohol consumption. Further ongoing support provided after the 12 weeks through a reunion session at the club and email prompts sent by club coaches at 6-weekly intervals. Participants also encouraged to meet up outside of the group to provide mutual support (either organized through the club or at an independent level).	Coach-led encouragement of banter (e.g., football-related, ironic or self-deprecatory jokes). Facilitation of humor helped men address serious or sensitive topics that they may otherwise be reluctant to discuss (e.g., weight gain).	Gender-responsive
11. Football in the Community (FitC)	Participants were recruited through a men’s homeless shelter and drug addiction service within the City of Liverpool (both of which were in partnership with the Everton Football Club FitC). Recruitment methods included face-to-face engagement, referral from service staff, word of mouth, and phone calls.	Delivered through the Everton Football Club’s FitC scheme. Intervention sessions were delivered in a local community football facility.	Sessions were delivered by a qualified FitC coach.	After completion of the intervention sessions, men were given the opportunity to enroll in the ongoing weekly activities of the broader Premier League Health program (e.g., football, circuit training and boxing). Participants discussed the factors that prevented them from attending sessions (e.g., economic, environmental, and social challenges).	Gender-responsive
12. HAT TRICK	Recruitment via the Rockets Hockey team (e.g., poster advertising, team website, newsletters, game day intercom announcements, information booths at home games), local media (e.g., print newspapers, television and radio broadcasts), email and print communication through local male-dominated community organizations, social media (e.g., Facebook, Castanet, Kijiji), poster advertisements in the community (e.g., coffee shops, ice hockey arenas, large hardware entities), word of mouth (e.g., through previous participants), and HAT TRICK website.	Delivered at the local hockey arena (home facility of the Rockets). Sessions did not occur on the ice surface, rather other spaces around the building (e.g., walkways, bleacher stairs, parking area, training facilities, nearby sports/community facilities).	Delivered by 2 male members of the research team (with degrees in sport and exercise science) and a certified group fitness trainer. Hockey team personnel and community experts were also invited as guest presenters for selected sessions. An interactive and informal learning style was used (e.g., group activities, competition) to enhance social connectedness and camaraderie. Men were also given a wearable device (i.e., Fitbit) to self-monitor their physical activity progress.	Gender-related elements that were incorporated into the program design included men’s preferences for activity-based approaches, self-monitoring and friendly competition, space for male camaraderie, normalized health-related practices, mobilized men in regaining fitness, and valued masculine activities and identities. Participants received a HAT TRICK “Playbook” that summarized key messages and provided extra resources. All resources incorporated a masculine look and feel. This included the use of dark color tones, images of average men exercising, language (e.g., “power foods”), and tone (e.g., “I don’t need to eat like a rabbit or live at the gym to improve my health.” All messaging was clear, positive, and direct, incorporating hockey-related themes and references to frame topics while emphasizing strategies that appeal to masculinities (e.g., independence, self-reliance, mastery).	Gender-transformative
13. He Pikinga Waiora	Cohort 1 was organized through a Christian church and social media platforms like Facebook, with participation open to both church members and non-members. Women were specifically engaged to facilitate the involvement of their partners. In contrast, Cohort 2 was organized through a local gym trainer’s personal and participant network. Both cohorts were chosen based on input from stakeholders during the co-design process.	The physical activity element for Cohort 1 was delivered through participant self-organization. The physical activity element for Cohort 2 was delivered through participant-identified means (e.g., phone, face-to-face, home visit). The nutrition element was delivered via a booklet.	Central elements of the program included cultural-centeredness, community engagement, systems thinking, and integrated knowledge translation. In addition to the co-design process, the intervention incorporated components from various lifestyle interventions (e.g., the Diabetes Prevention Program). Cohort 1 was delivered by a Tuakana (senior mentor) who was also a participant. Cohort 2 was delivered by a Kaiarahi (guide or community health worker).	Facebook groups and information booklets were used in cohort 1. Not otherwise described.	Gender-responsive
14. Heart of Hypertension	Street outreach and marketing through direct contact (e.g., text messages) or flyers. In addition to established relationships from previous projects and participation in HT’s annual community health screening events for men.	Health screening and pilot testing were carried out at the HT facility.	The planning team involved researchers (who had studied the beliefs and lifestyles of young African-American men or research in community/sport development and cardiovascular fitness), a clinical social worker, research assistants, and the Hayes-Taylor Memorial YMCA (HT), situated in a predominately African-American neighborhood. The exercise sessions in the intervention were led by African-American instructors and involved cardio drills, circuit training, agility exercises, and power yoga. Participants had also suggested the intervention use sports other than basketball (e.g., martial arts, cycling, conditioning, kick boxing, water aerobics, and dance).	Regarding dinners, men received recipes, related information, and a short food preparation demonstration. Participants also received a study binder with intervention handouts, recipes, and related materials. Focus group participants suggested the program should avoid a classroom atmosphere and encourage group interaction. Participants also felt the program logo should represent placing value of heart health through the collaboration of African-American men of all sizes. They also created the name *Heart of Hypertension.*	Gender-responsive
15. HEYMAN	Participants were recruited via flyers, social media posts, media release (i.e., local newspaper, radio station, magazines). Participants from the previous participatory research who were interested in being contacted about future health programs were also invited via email.	Delivered at the University of Newcastle (for face-to-face sessions) and self-guided delivery at home.	Participants were provided with the intervention materials (e.g., exercise trackers, resistance band) at baseline and instructed to use them over the 3-month intervention period. Face-to-face sessions were delivered by two male researchers from the same age demographic (i.e., 18–25 years).	Recruitment materials emphasized young men’s individual preferences and addressed perceived motivators and barriers (e.g., fitness and strength). Graphic design and branding also reflected young men (e.g., using images of young men) as well as other male engagement strategies (e.g., sensitive use of humor). Private Facebook groups were provided to facilitate social support, notify when new resources were added to the website, and reminders for upcoming sessions.	Gender-transformative
16. Hockey FIT	Participants were recruited using a variety of methods, including team email blasts, social media (e.g., Twitter), posters, traditional media advertisements (e.g., newspaper, TV, radio, community magazines), and the Hockey FIT website.	Sessions were run at the hockey team home arena or at a local fitness club.	The program was delivered by two trained Hockey FIT coaches: a Head Coach and an Assistant Coach. It featured flexible delivery, allowing the coaches to adapt the program according to their individual strengths and weaknesses, align with participant personalities, and accommodate the specific environments of each session (e.g., arenas, community centers, health club facilities).	Sessions were designed to appeal to men, including elements of friendly competition and encouraging banter, humor, and peer support. Participants were encouraged to use the Hockey FIT social network to share resources, stories, ask questions, and keep in touch with other participants and coaches.	Gender-sensitive
17. Kerala Smoking Cessation	Participants were recruited from 4 (of 12) community development blocks in the rural Thiruvananthapuram district, Kerala.	Initial survey delivered in house-to-house visits. A combined approach was used for the intervention (i.e., phone counseling, face-to-face interviews). Priority given to in-person.	A combination of four approaches utilized (i.e., distribution of educational materials, invitations to key personnel in the locality to attend the medical camp and counseling, conduct of medical camp and group counseling, and individual counseling at four time points). Initial survey and anti-tobacco leaflets delivered by trained female Accredited Social Health Activists. Counseling was delivered by health professionals and medical social workers. Healthcare providers, local administrative heads, health workers, and medical doctors were also requested to support the program.	All participants were given multi-colored anti-tobacco leaflets with information on smoking hazards (e.g., health concerns, passive smoking for women and children). The leaflets also discussed the importance of “role modeling” by men in the cessation of tobacco use in the community.	Gender-sensitive
18. MAN vs FAT Football Program	MAN vs FAT website. Not otherwise described.	Delivered at various local football facilities around the UK. Participants are able to choose a location convenient to them.	To promote men’s uptake and effectiveness, the program incorporated sport-based physical activity, social support, contact with other men, gender-specific advice, access to self-monitoring tools, and a competitive approach toward weight loss. Each team also nominated a captain (liaise between coach and team, encourage camaraderie, motivating and rallying the team). Men identified the appeal of sport, male-centered approach and masculine environment, competition, level playing field, being part of a team, camaraderie, accountability, and interaction with likeminded men as positive factors driving recruitment, program effectiveness, and retention.	The program fostered a male or gender-sensitive environment by creating a shared understanding among participants that they were there for similar reasons. This environment allowed for friendly banter, light-hearted jokes, and a competitive spirit. Participants were encouraged to join team WhatsApp groups and online forums for support, with a focus on sharing male-specific issues with other men.	Gender-responsive
19. Meaning-Centered Men’s Groups	Recruitment efforts included hard-copy flyers distributed in community locations, soft-copy flyers on Web-based community newspapers and bulletin boards, dissemination through community partners and collaborators, direct delivery to in-person addresses, media interviews, and promotion at local health and wellness fairs and community events.	Delivered in community settings, not otherwise described.	Delivered by two male facilitators (the project principal investigator and a community-based social service provider). Group sessions focused on interpersonal and intrapersonal transitions associated with retirement (i.e., meaning in creativity, meaning in experiences, meaning in attitudes, and meaning in life and generativity).	The program was marketed as a “men’s group dealing with adjustment to retirement” rather than a “psychotherapy group” to help normalize concerns about retirement. The group format promoted social discourse, camaraderie, and support among members. It included a “go-around” where each participant shared their thoughts, personal stories, and reflections, and offered support to others. Participants were also encouraged to stay in touch after the sessions ended. Sessions were based on themes from Frankl’s *The Doctor and the Soul*, covering purposeful activities, exercise, relationships, and societal expectations. In addition, handouts were provided to help participants find meaning and clarify their personal thoughts, feelings, and experiences.	Gender-responsive
20. Men on the Move (Ireland)	Recruitment occurred across eight counties in Ireland. Otherwise not described.	The program was delivered at 12 intervention group sites and 13 comparison-in-waiting group sites. Otherwise not described.	The program was funded by the Irish Health Executive Service as a gender-specific, community-based “beginner” physical activity intervention. Men on the Move is largely based on Football Fans in Training.	Some flexibility was afforded to ensure core components were achieved in line with participants’ needs.	Gender-sensitive
21. Men on the Move (USA)	Men were recruited though the UM Comprehensive Cancer Center Community Outreach Program’s Men’s Fellowships breakfasts. Flyers were disseminated at the breakfasts and previous breakfast participants were sent emails with information on the study.	Baseline physiological assessments were done by nurses at UM. Workout sessions were delivered at a common location, otherwise not described.	Four of the five trainers were African American, three were within the eligible age group (i.e., ≥35 years), and all had previously trained with male populations. Workout sessions provided men with structured opportunities to exercise and included activities that promoted balance, flexibility, strength, and conditioning. The trainers designed these workouts based the fitness and diversity in their groups.	Men completed regular physical activity logs during the intervention and provided open-ended feedback during the impact assessment at the end of the program. Participants were given additional resources including a schedule of fitness classes offered in the area, stretches and exercises to do at home, and a list of relevant websites. Men were also given the contacts of other group members and were encouraged to exercise with each other between sessions (facilitating access to social support from male peers).	Gender-responsive
22. Mind Resilience Program	Men were recruited through partnerships with community groups. Engagement strategies tailored to men’s needs (e.g., immediate need to make rent payments) and motivations (e.g., self-improvement) in ways that do not evoke self-stigma.	Delivered in various community-based settings including enjoyable and action-oriented activities.	Flexibly delivered and modified to participant interests (e.g., football, photography, gardening, drumming). Practical activities, focused on male identities (e.g., gender norms), trust-building, social support, and insight-based coping strategies to address self-stigma. A safe space for the coping strategies program.	Built trust and belonging with shoulder-to-shoulder discussions. Emotional issues were introduced with a light touch and acceptable language such as “managing” and “down” and “focused” helped to build trust.	Gender-transformative
23. MOCHA Moving Forward	Men recruited through free public events (e.g., Jazz festival), information tables at select community agencies, social media, program website, billboards, posters (e.g., barbershops), and local radio talk shows.	Community-based settings in Springfield, MA. Not otherwise described	The program utilized a peer delivery model where MOCHA Steering Committee members were trained as community health workers and group facilitators to deliver the MOCHA/+ curricula. The content was specifically tailored to address the needs of African-American men in the greater Springfield area, taking into account the unique social context and socio-cultural drivers of their health behaviors.	Culturally and gender-responsive materials (e.g., digital stories and narratives).	Gender-transformative
24. Offload Rugby League Program	Recruitment through sports clubs and select organizations. Not otherwise described.	The program was delivered in three types of locations: (a) rugby stadia, where men attended without prior booking; (b) select locations such as workplaces, prisons, and sheltered accommodations, where attendance was voluntary but restricted to those within the host organization; and (c) colleges, where attendance was compulsory and participants were selected by the college, addressing recruitment challenges faced by the club.	The program was delivered by a foundation staff member alongside either a former rugby league player, coach, official, or mindfulness specialist with lived experience of mental illness, including depression, anxiety, or suicidal ideation. The sessions featured interactive content, such as physical activity tasters and pub quizzes, aligned with themes like stress management techniques, mindfulness, and life balance.	The program employed sport-related analogies and metaphors, referring to facilitators as “coaches,” participants as “players,” and groups as a “squad.” Male-relevant language, often incorporating sporting terms like “coping,” “goal setting,” and “winning mindset,” was used throughout. The facilitators also used humor and language that resonated with men, drawing on shared tacit knowledge from their own lived experiences.	Gender-transformative
25. Power Up for Health	Recruitment included flyers distributed in local community (e.g., recreation centers, libraries, barbershops), emails through community organizations, newspaper advertisements, letters sent from primary care physicians in target neighborhoods, social media, community presentations, and targeted mail to men living near each of the recreation centers.	Community recreation centers in local neighborhoods.	The NDPP was delivered in group settings by male lifestyle coaches who complete a 2-day training on the curriculum by NDPP-certified master trainers.	Content utilized photos, examples, and quotes that appeal to men (e.g., statistics on erectile dysfunction in “quick facts”).	Gender-responsive
26. POWERPLAY	Recruitment was conducted using tailored poster and health screenings within the workplace.	Male-dominant workplaces.	Each challenge was themed to foster friendly competition between teams organized by the workplace. In the first challenge, participants used pedometers to track their daily step counts and received educational materials on topics such as staying active at work, healthy eating on the go, stress management, making healthy drink choices, and maintaining physical activity. The second challenge, known as the POWER PLAY-OFF Challenge, was designed as a “virtual” hockey game, emphasizing the accumulation of physical activity minutes rather than steps, and required meeting several pre-determined healthy eating goals.	All resources and materials were designed to be gender-sensitive, featuring a masculine look and feel with clear messaging about physical activity and healthy eating. They utilized gain-framed messaging and man-friendly language, imagery, and examples, such as staying active to keep up with kids and being a provider for family.	Gender-responsive
27. Premier League Health (PLH)	Recruitment included advertising and outreach (e.g., events at clubs) delivered through partners.	Delivered in football club facilities and community venues.	Flexible delivery health information tailored to participants needs and interests that leverage club branding to increase engagement. Facilitators included health trainers, allied health professionals, and club staff wearing branded clothing.	Trainers were flexible and responsive in their content delivery with an aim to be informal, familiar in their communication.	Gender-sensitive
28. QuitNow Men	Men were recruited using social media (Facebook and Twitter) and online classified advertisements (Kijiji, Craigslist, and Castanet).	Men-centered smoking cessation website allowed for anonymity and convenience (in comparison to face-to-face counseling).	Offered a variety of resources and practical strategies to increase the likelihood of a quit attempt.	Utilized masculine images, direct language, and content including an interactive video drama. Communication included male-friendly language, strong, positive messages to promote change (e.g., “Put these tactics and tools to good use to get the job done”). Connected positive identities, such as being healthy and strong, with being smoke free, and included men’s stories about quitting to show common challenges and create a community of mutual help.	Gender-responsive
29. RUFIT NZ	Recruitment occurred via respective rugby club’s fan registries (e.g., Facebook pages, supporter mailing lists, newspaper ads/articles) and Māori-specific networks (e.g., Māori meeting house, word-of-mouth, and Māori TV and radio).	Delivered via professional rugby franchises.	All RUFIT-NZ trainers were qualified strength and conditioning trainers involved with the respective rugby clubs. Nutrition-based components were delivered by the clubs’ nutritionists or qualified dieticians.	Communication encouraged a sense of teamwork and collecting “wins,” including simple messaging and practical information.	Gender-sensitive
30. SHED-IT	Recruitment was conducted using media articles (e.g., newspaper, radio) and workplace notices.	Self-guided delivery to allow for autonomy.	The SHED-IT programs were designed to accommodate men’s preference for independent lifestyle programs by avoiding face-to-face contact. Resources promoted nine evidence-based, theory-driven weight loss messages. Men in the SHED-IT Online group received a user guide for the website (developed by the research team) and were instructed to use the online food and exercise diary on the freely available CalorieKing (Australia) website. In addition, they received seven feedback emails regarding their food and exercise diary entries.	Communication in the program included humor, anecdotes, and strategies tailored to men, such as incorporating steps taken on the golf course. It featured statistics and research from men-only studies and included images of men. The content used male-friendly examples, such as achieving energy balance without entirely eliminating “luxury” foods like the occasional beer.	Gender-sensitive
31. Sheds for Life	Invited men from existing Men’s Sheds to receive a comprehensive health check as a “hook” to engagement.	Delivered free of charge in a non-clinical, male-specific, safe, and familiar environment. Group activities allow for a sense of social support inherent in Sheds. Researchers visited each individual Shed in advance of the program commencing, to brief Shedders on the program, to build a sense of rapport and trust, and to assess the Shed environment’s suitability to participate in the program.	Used an informal and interactive delivery style to maximize engagement and enjoyment. Fostered an environment of openness and peer support between participants, utilizing a non-competitive and relaxed environment where participants engaged at their own pace. Health-related information and content tailored to each Shed to provide a sense of autonomy and control for Shedders. Used non-typical health related components such as digital literacy and CPR as additional hooks to engage those less reluctant to sign up to a more conventional health program.	Communication was relaxed, encouraged banter, and individual emphasized choice and control.	Gender-transformative
32. SHIFT	Participants were recruited using posters within their workplace.	Delivered within transport sites where men worked.	Incorporated group-based discussions and activities. Technology-based tracking with personalized feedback.	Co-delivered by individuals who were HGV drivers and driver trainers.	Gender-blind
33. Sons of the West	Participants were recruited through community groups and notice boards, word of mouth, recommendations from health practitioners, and media/online advertisements.	Community-based settings utilizing branding of AFL team.	Information-based presentations were delivered by various partner organizations with expertise in health topics, including nutrition, mental health, cancer, preventative health screenings, cultural inclusion, alcohol, gambling, and promoting gender equity. Exercise groups were led by local practitioners such as personal trainers and exercise physiologists. Provisional psychologists on postgraduate placement also attended each site to engage with participants.	Communication included culturally-themed content, and male-specific health topics. Speakers included men with lived experience who explicitly discussed masculinity.	Gender-transformative
34. Truckin’ Healthy	Direct contact to transport organizations known to members of the project team and workplaces identified as members of the Australian Trucking Association’s *TruckSafe* program	Transport industry workplaces in south-east Queensland.	A total of seven workplace health promotion interventions were developed. These included displaying healthy eating posters in the workplace, installing a healthy options vending machine, and supplying free fruit to drivers. A 10,000 steps workplace challenge was also introduced, along with healthy eating and physical activity toolbox talks. Health messages were distributed to drivers through their payslips, and a dedicated Truckin’ Healthy Facebook webpage was created.	NR	Gender-blind
35. ViSiT	Recruitment was conducted through the clubs’ websites, Facebook pages, and via an email sent from the club to its members. The invitation was signed by the club director, representatives from the non-profit sport organization, and the primary investigator.	The sessions were held in facilities connected to the club, either at the club stadium for the football club or at a gym owned and used by the club for the ice hockey club.	Group-based sessions were delivered by a project manager from the Östergötland Sports Federation in collaboration with a public health practitioner who planned and conducted the lessons. Several men reported that the intervention provided them with a new perspective, breaking their existing behavioral patterns. They also noted that their transformed way of life had a positive impact on those around them, such as colleagues.	The program used male-friendly language, such as “fight hard and succeed,” and encouraged open discussions along with a competitive spirit.	Gender transformative

## Discussion

### Participatory Methods in Men’s Health Promotion

The current scoping review findings deepen understandings about the ways in which participatory action methods have been employed, and how they can be utilized in men’s health promotion research. This is a critical step forward given the variability in operational definitions challenging researchers to decide what elements might most effectively count as participatory action. For instance, while most studies reported the use of co-design initiatives, this was loosely defined and used interchangeably with co-production, co-creation, co-development, as well as concepts such as “collaboration,” “working in partnership,” and “engaging end-users, stakeholders, and/or consumers.” The inconsistency of terminology and associated diverse approaches have garnered much debate in health research about the level at which end-users can and/or need to be engaged in the intervention development processes (Clarke et al., 2017; Pearce et al., 2020; Slattery et al., 2020).

Beyond the semantics of participatory action methods there is much to be learnt from the patterns and diversity evident in the synthesis of the 35 programs featured in this review. Foremost, it is fair to say that participatory action research has been and is perhaps for evermore altered by the COVID-19 pandemic, wherein virtual participation (and program delivery) predominates (Oliffe et al., 2023). Deviating from traditional group-based, in-person programs, many programs necessarily pivoted to 100% online delivery with the pandemic and all that has followed. Herein, the specificities of those interactions can be understood as shifting in ways that might facilitate or incumber the involvement of stakeholders in the design and delivery of men’s health promotion programs. While new participatory action methods and models for men’s health promotion should be rooted in community-based program principles, with a focus on power and empowerment, the mechanisms and means for virtual involvement demand pragmatic diversity (and perhaps a new paradigm) for doing that process work.

As contributors, men were involved at a consumer control level in very few of the reviewed studies (i.e., researchers were involved only at the request of the end-users who create, undertake, and disseminate the results of the intervention) (Bergin & Richardson, 2021; Joseph et al., 2023; Valdez et al., 2021). Rather, they were more likely to be engaged at the consultation (i.e., seeking stakeholders’ views and using them to influence program development) or collaboration stage (i.e., continuous partnership between stakeholders and researchers) (Boote et al., 2002, 2011). Indeed, campaigns largely engaged men at the evaluation stage post-development, often in the form of feasibility or acceptability measures (e.g., focus groups, interviews, surveys). Further, few studies included men at the sharing stage of the process, and of those that did, none engaged men beyond a consultation role (Jayakrishnan, Uutela, et al., 2013; Warbrick et al., 2020) despite the possibility of greater dissemination engagement and wider public awareness (Brett et al., 2014; Constantin et al., 2022). At any phase, roughly half of the programs did not go further than an initial one-off consultation with little detail provided concerning how the information gathered was used to inform the co-design process. Overall, our findings highlight the lack of strategic and sustained contributions from those that are supposed to benefit from the interventions, a trend which presents missed opportunities to achieve the most relevant and meaningful design and implementation outcomes (Boote et al., 2011; Schulenkorf, 2010, 2012). It has long been recommended that end-users be involved in all stages of intervention development to help increase their capacity and agency within the process and to enhance the effectiveness of the program and associated dissemination (Boote et al., 2011). That said, we recognize that priority groups of end-users often face systematic disadvantages and suggest that considerations for minimizing participant burden and/or providing compensation are key to the feasibility of participatory action research methods. Where possible, we recommend identifying and working closely with key stakeholders (e.g., industry, policymakers) to leverage and sustain the design and delivery of men’s health promotion programs.

Some programs such as Aussie-FIT or Euro FIT were developed based on the literature and existing program design models (i.e., FFIT) (Quested et al., 2018; van Nassau et al., 2016). As such, these programs are likely built on “secondary participation” where diverse perspectives and evidence are considered through indirect and passive means. Secondary participation can be risky; for instance, in the case of FFIT, the program itself only engaged men in a consultation capacity at the refining and evaluation phases of development, leaving participation as an end-point rather than a program design process. Relatedly, there are numerous complexities regarding contextual and cultural factors when adapting programs that men may be able to provide valuable insight to (Copeland et al., 2022). This may be critical in the context of commercial and structural determinants of health, where profit-driven neoliberal sectors shape public health debates as well as emergent health trends (de Lacy-Vawdon et al., 2022). Taken together, increasing the participatory action of men and relevant stakeholders holds promise to effectively design meaningful programs, and increase capacity, ownership and sustainability potential for men’s health promotion programs. The sport-based intervention model has dominated men’s health promotion interventions and its success at engaging men and achieving health outcomes globally is evident (Timm et al., 2024). However, as we look to the next generation of men’s health promotion interventions addressing gender and health inequities, participatory action methods that reside outside traditional masculine arenas (i.e., sports) will be critical to scaling and sustaining innovative interventions.

Finally, the current paradigm of men’s health promotion programs is built on time-limited funding, highlighting a critical limitation in long-term program development and evaluations. All studies reviewed were at least partially supported by limited-term grants from government, university, and non-profit organizations. Programmatic funding and renewals would ease pressures on program leaders and staff and build sustained participatory actions with partners. This context underscores the necessity of stable, long-term funding solutions to ensure long-term success and the impact of men’s health promotion efforts (Oliffe et al., 2024).

### Gender Responsiveness in Men’s Health Promotion Programs

Study findings revealed considerable diversity in how interventions were targeted to and tailored for men. Overall, there has been a reliance on traditional masculine norms as the centerpiece by which men’s health behavior changes are explained (and claimed) as antiquated, shifting, and/or sub-altern. In referencing gender-transformative efforts, the hegemony of traditional masculinity has prevailed to the point where we are likely blind to generational and life course changes in men’s masculine ideals. While some programs in the current review were complicit in sustaining traditional masculinity as a lynchpin for marketing and/or *the* pivot for lobbying change through tailored interventions, there is a need to consider “our” collective knowledge of contemporary masculinities to inductively derive and address gender-responsive approaches. It seems entirely worthwhile knowing the practices and needs of discrete sub-groups of men regarding their health. Young men, for example, have grown up in e-spaces with reports suggesting that they are increasingly isolated, lonely and unable to connect socially (Barreto et al., 2021). To be gender-responsive in this context demands knowledge of the “problem” and feasibility for a “remedy” to address challenges for contemporary masculinities. Said another way, gender responsiveness needs to understand and address young men’s isolation rather than map the problem or solution to shifting or relying on traditional masculine norms. Only one third (*n* = 12; 34%) of interventions were evaluated as gender-transformative, suggesting that there is still much work to be done in this regard.

While the GRAS provides an important frame, its application to men’s health promotion programs poses several challenges. The shortfall with transformative approaches is that they assume a problem orientation, often with traditional masculine norms as the baseline, and needle to be moved. The predominance of gender-sensitive programs has similarly nestled men’s health behaviors as flowing from traditional masculinities, and the lines between gender-sensitive, gender-specific, gender-transformative and gender responsive suffer the same fate—with traditional masculine norms as the baseline. In the strength-based spaces of masculinities, the healthful twist on traditional masculine norms assumes the interventions need to induce men’s reformulation of risky practices. These programs are reactive for the most part rather than responsive to the sub-group that are espoused as being tailored and responsive to. Herein, the emphasis of transformative interventions on individual responsibility for self-health have been assumed to stem from altering men’s behaviors. Social determinants of health predominantly featured as an inclusion criterion (e.g., target demographics) rather than an intervention outcome in terms of addressing health inequities at a population health level. These findings indicate strong interest in addressing the intersections of gender and culture in segmenting end-user sub-groups. This approach passes as gender-responsive in line with a social determinants of health frame, and promissory notes for focussing on addressing men’s inequities. Within these contexts, the emphasis however remains individual behavior change while the structural determinants of health rendering men to live in marginalizing conditions need attention (Heller et al., 2024). Clearly, men’s agency is, but cannot solely be, the core business of men’s health promotion programs. For the reviewed studies, and men’s health promotion more broadly, it can be difficult to estimate the broader socio-cultural impacts that programs have on masculinities, and the structural determinants of health. Yet, well-designed consumer-led programs within communities might be understood as contributing to, or stemming from, a net positive shift in our collective understanding of the interplays between gender and health.

Several programs targeted men from diverse backgrounds that often incorporate specific cultural or racial contexts which may not be fully captured in traditional gender-responsive definitions. By acknowledging and addressing these intersections, health promotion programs can be inclusive and effective. That said, significant effort has been given to providing rich descriptive data of the experiences and challenges of men from diverse backgrounds. However, few studies are able to bridge this work to effectively address underlying social determinants; Lomas (1998) highlights this disconnect, showing that 90% of the research is focused on providing groundwork that richly describes the “problems.” This gap raises questions about the feasibility of developing tailored programs for every inequity, especially in a post-COVID-19 public health care system that is financially strained (Oliffe et al., 2021). Herein, the amalgamation of programs and services for men living in marginalizing conditions enhances the reach and impact of health programs, making them more sustainable in the long term. This approach is increasingly relevant in the current economic climate, where public health care systems are reeling post-COVID. As evidenced by the studies in the current review, men can and do meaningfully adapt *their* health promotion programs when programs are collaboratively or consumer controlled (e.g., Bergin & Richardson, 2021; Robinson et al., 2015). Recommendations for practitioners and researchers designing health promotion programs for men are provided in Table 3.

**Table 3:** Recommendations for Practitioners and Researchers Designing Health Promotion Programs for Men.

**Designing gender-responsive health promotion programs for men**
• Ensure clarity and consistency in defining and reporting participatory action methods
• Design participatory processes that engage men and key stakeholders at all stages of intervention development, from initial concept to dissemination
• Recognize and address power dynamics to ensure equitable partnerships between researchers and end-users
• Establish partnerships with industry, policymakers, and community organizations to enhance the sustainability of interventions
• Provide pathways for end-users to transition from consultation to collaboration and consumer control roles
• Engage men from diverse backgrounds throughout the intervention process to ensure cultural relevance
• Incorporate contemporary understandings of masculinities to ensure interventions are relevant to diverse sub-groups of men
• Move beyond problem-oriented frameworks that focus solely on altering individual behaviors and instead address the broader socio-cultural and structural determinants of health
• Address intersectional issues by tailoring interventions to the unique needs of different sub-groups
• Consider creative and novel approaches that accommodate socio-political and economic contexts that influence men’s health behaviors and outcomes
• Prioritize gender-transformative approaches that address the underlying social determinants of health

### Strengths and Limitations

This is the first review to explore participatory action methods and the operationalization of gender-responsive interventions for men, and thus significantly contributes to a synthesis and opportunities for advancing men’s health promotion research. The importance of this work is underscored by well-documented evidence concerning the challenges with recruiting, engaging, and sustaining men in health promotion and illness-prevention initiatives (Bell et al., 2023; Bottorff et al., 2015; Howell et al., 2023; Sharp, Spence, et al., 2020) and the promising research highlighting the importance of co-design approaches (Galdas et al., 2023; Kinsman et al., 2021). In addition, this review included a rigorous methodology with a comprehensive search strategy, to include the most relevant published studies.

Despite these important strengths, our review has some noteworthy limitations. First, because we restricted our search to published research written in English and excluded gray literature (e.g., government and/or policy statements, stakeholder/end-user reports), we compromised our global understanding of some structural determinants of health. Our search strategy was also limited due to the considerable variability of terms around “co-design” and “participatory approaches,” which may have obscured some applicable research. Given the heterogeneity of interventions, we cannot confidently attribute the use of specific participatory action methods or gender-responsive approaches to improved study outcomes. Future research should aim to scale and sustain gender-responsive programs inclusive of formally evaluating specific outcomes (e.g., engagement/retention, health behaviors/outcomes). Finally, programs specific to sexual or oral health were excluded as it was considered that these concepts were too nuanced to be adequately covered in the search strategy.

## Conclusion

This scoping review examined the use of participatory action methods and gender responsiveness in men’s health promotion programs. We surmise that participatory methods do not guarantee gender responsiveness when designing health-promotion programs for men. Yet, participatory methods and gender-responsive approaches can work synergistically to create programs that are informed by the needs and perspectives of men with sensitivities to the ways in which masculinities influence health behaviors and engagement (S. Robertson et al., 2013; Sharp, Spence, et al., 2020). Researchers must expressly consider how to utilize gender-transformative approaches to address contemporary health inequities that do not rely on traditional masculine norms.

## Appendix

**Table:** Search Strategy.

#	Searches	Results
1	exp Male$/	9,329,496
2	(men or man).ti, ab,kw.	897,603
3	1 or 2	9,521,138
4	Preventive Health Services/	14,439
5	exp Health Promotion/	84,870
6	exp Primary Prevention/	180,395
7	exp Mens Health/	2,168
8	(health$ adj (promot$ or lifestyle or program$ or intervention$ or campaign$ or initiative$)).ti,ab,kw.	108,186
9	(lifestyle adj ((change$ adj behavio?r) or intervention$)).ti,ab,kw.	9,441
10	((well-being or wellbeing) adj1 promot$).ti, ab,kw.	1,319
11	or/4-10	362,115
12	exp Community-Based Participatory Research/	5,951
13	exp Consumer Participation/	47,666
14	((pre or co or participat$ or collective or consumer or stakeholder) adj (design or creat$ or research or produc$ or method$)).ti,ab,kw.	20,263
15	((user or human) adj (center$ or center$ or participat$ or engage$ or involve$)).ti,ab,kw.	5,483
16	(((community or experience) adj based) or (community adj (engagement or participation or involvement)) or ‘action research’ or ‘needs analysis’).ti, ab,kw.	88,949
17	((sex or gender$ or m?n or male) adj (aware or appropriate or tailor$ or sensiti$ or focus$ or transformative or mainstreaming or specific or center$ or center$ or responsive or informed or involve$)).ti,ab,kw.	47,901
18	or/12-17	201,227
19	3 and 11 and 18	6,561
20	limit 19 to english language	6,382
21	20 not (exp animals/ not humans/)	6,353
22	limit 21 to male	6,126
23	22 not (infant or child$ or adolescent$ or youth or teen$ or juvenile).ti,ab,kw.	4,286
24	limit 23 to yr=“2013 - 2023”	2,575
